# Local Anæsthesia

**Published:** 1862-10

**Authors:** 


Local Anæsthesia.—Mr. Fournie recommends for the induction of local anæsthesia, a mixture of equal parts of glacial acetic acid and chloroform. He states that complete insensibility of the part may be obtained in five minutes.

